# Self-Efficacy Beliefs Are Associated with Visual Height Intolerance: A Cross-Sectional Survey

**DOI:** 10.1371/journal.pone.0116220

**Published:** 2014-12-30

**Authors:** Eva Grill, Florian Schäffler, Doreen Huppert, Martin Müller, Hans-Peter Kapfhammer, Thomas Brandt

**Affiliations:** 1 Institute for Medical Information Processing, Biometrics and Epidemiology (IBE), Ludwig-Maximilians Universität München, Marchioninistr. 17, 81377 Munich, Germany; 2 German Center for Vertigo and Balance Disorders, Ludwig-Maximilians Universität München, Marchioninistr. 15, 81377 Munich, Germany; 3 Hochschule München - University of Applied Sciences, Department of Applied Social Sciences, Am Stadtpark 20, 81243 Munich, Germany; 4 Institute for Clinical Neurosciences, Ludwig-Maximilians Universität München, Marchioninistr. 15, 81377 Munich, Germany; 5 Department of Psychiatry, Medical University Graz, Auenbruggerplatz 31, 8036 Graz, Austria; Technion - Israel Institute of Technology, Israel

## Abstract

**Background:**

Responses to height may range from indifference to minor distress to severe symptoms of fear of heights (acrophobia); visual height intolerance (vHI) denotes the whole spectrum of symptoms. Although there are options to manage vHI, only a small part of persons affected by vHI are willing to seek professional help or confront their problem. Purpose of this study was to determine if persons with vHI, specifically those who show avoidant behavior towards heights (avoiders), score lower in their general self-efficacy (GSE) than those who confront vHI (confronters).

**Method:**

Cross-sectional survey in 607 individuals living in the urban region of Munich, Germany, using a mailed questionnaire on presence or absence of vHI, confronting or avoiding behaviour, and GSE.

**Results:**

Of all participants (mean age 53.9, 50.3% female), 407 reported life-time presence of vHI. Participants with vHI had a mean GSE score of 31.8 (SD 4.3) points (participants without vHI: 32.5, SD 4.3, p  = 0.008 for difference). Among individuals with vHI, 23% reported confronting behavior. Confronters were significantly younger (p<.0001, 50.2 vs. 55.7 years), more likely to be female (p  = 0.0039, 64.3% female), and had a higher GSE score (p  = 0.0049, 32.5 vs. 31.1). Associations remained significant after multiple adjustment.

**Conclusions:**

Our study provides evidence for the association of GSE and vHI. These findings may have consequences for strategies of alleviation and therapy of vHI.

## Introduction

The visual perception of heights generally elicits postural imbalance [1]. This is a common physiological response. Individuals, however, react differently, and their responses may range from indifference to minor distress to severe symptoms of fear of heights (acrophobia) [2]. Fear of heights is classified by the ICD-10 [3] and DSM-V [4] criteria as a specific phobia. It has a life-time prevalence of 3.1% to 6.4% [5]–[8]. The more general term “visual height intolerance” (vHI) comprises varying signs of distress and vertigo in the presence of heights; vHI affects almost one third of the population [2], [9]. Thus, one might define three distinct conditions: physiological postural imbalance that affects everyone, vHI, and acrophobia as a specific phobia with symptoms of a panic attack. Two of the three conditions, vHI and acrophobia, might warrant treatment. While acrophobia can be regarded as a significant clinical problem, the presence of vHI might reasonably be the motivation to participate in non-clinical training programs, arguably for those individuals who are interested in mountaineering despite their condition. This idea of a clinical distinction between vHI and acrophobia based on severity and relevance, however, was recently contradicted by findings from a population-based study where 23% of individuals with vHI reported symptoms that reached the intensity level of panic attacks [6]. Also, this study established vHI as an indicator for overall anxiety such as social phobic and hypochondriac fears [6]. This makes the case for regarding vHI as the umbrella term for a continuum of reactions to height stimuli, and for considering acrophobia as its most extreme manifestation.

The symptoms of vHI are manifold. Their consequences for the individual vary depending on numerous aspects, e. g., on the specific trigger, on the type of visual stimulation, on the individual constitution, and on the individual understanding of danger and safety. Affected individuals may report restrictions in their daily life [10] which can appear to be severely disabling and have major consequences for quality of life and functioning [5].

Individuals with acrophobia are known to overestimate height and the danger associated with it [11], [12]. A subclinical balance dysfunction may increase body sway during height exposure [13]–[15] and therefore lead to increased fear of heights. Moreover, individuals who largely depend on visual clues for body stabilization might particularly be predisposed for acrophobia [14], but this is responsive to training. Consequently, various training approaches make use of the observation that frequent exposure to heights reduces fear [16], [17]. In 1771 Goethe was probably the first to report that repeated exposure to height was a valuable therapy for acrophobia, which he had experienced atop the Strassburger Münster [1], [18]. This was later developed into a behavioral therapy concept of self-directed contact desensitization [19], [20] or implosion therapy (flooding) after Marks and Gelder [21].

Since then, exposure to heights as a therapy approach, also using virtual reality environments, has been extensively tested. Besides the models relying on perceptual and visuo-vestibular recalibration other factors such as cognitive and learning models can be useful for therapy [22]. Among those, the concept of self-efficacy has gained a prominent role. Social cognitive theory defines self-efficacy as the self-understood ability of individuals to deal with future situations and control potential threats [23], [24]. It was shown that successful cognitive behavioral therapy for anxiety works by changing self-efficacy beliefs [25]. According to this concept, threat is not an absolute value but results from a mismatch between one’s own coping abilities and the environment. To put this into the context of vHI, threat is experienced only when the external danger, for example, falling from an exposed mountain ridge, is felt to exceed the individual’s perceived skills.

On the basis of qualitative interviews with 18 individuals we recently posited the existence of two different coping typologies of persons with vHI: confronters and avoiders [10]. Confronters mentally anticipated their individual vHI scenario. They reported that they deliberately expose themselves to trigger situations or plan to participate in height training. In contrast, avoiders tried to skirt around heights or ignore an acute situation, e.g., by averting their gaze or closing their eyes. Avoiders either refused height training or had never consulted a doctor for this condition. Although the severity of symptoms or the amount of perceived danger does not seem to be a predictor of avoidance, perceived self-efficacy is [11], [26]–[28]. Since perceived self-efficacy was shown to be a determinant of avoidant behavior in acrophobia [26], it seems reasonable to assume that persons with vHI, specifically those who show avoidant behavior, score lower in their self-efficacy beliefs.

It could be shown that only a few individuals with vHI had already contemplated or completed height training [2]. In this regard, vHI aligns with specific phobias where only a minority of affected persons actively seeks professional help [29]. A focus on self-efficacy could potentially make such individuals more accessible to therapy.

The objective of this study was to examine the association between perceived self-efficacy and visual height intolerance. Specifically, we wanted to determine whether low self-efficacy is predictive of avoidant behavior in persons with vHI.

## Materials and Methods

### Study design and data collection

A cross-sectional survey was conducted in a sample of members of the German Alpine Association living in the urban region of Munich, Germany. The German Alpine Association (DAV) is a mass organization with almost 1,000,000 members. Its main focus is mountaineering and climbing, but it is also engaged in environmentalist issues, family programs, and holiday camps for children. Membership is rather common in southern Germany, even among persons with urban lifestyles. The DAV has 165,000 members in the respective area (DAV, personal communication). This corresponds to about 1% of the population. In May, 2013 a personalized letter along with a questionnaire was sent to 1,166 DAV members who had responded to a previous survey and had consented to be contacted again for further studies. A pre-paid response envelope addressed to the sponsoring institution was enclosed. Eligible participants had to be at least 18 years of age. Approval of the Ethics Committee of the Medical Faculty of the Ludwig-Maximilians University in Munich was obtained prior to starting the study. All participants provided their informed, written consent.

### Measures

The primary outcome was determining the presence or absence of self-reported lifetime vHI. Moreover, the percentage of individuals with vHI, who had consulted a physician and/or done height training was calculated. In line with previous studies [2], vHI was defined by the question “Have you already experienced visual height intolerance, an unpleasant feeling caused by visual exposure to heights?”. The secondary outcome in persons with self-reported lifetime vHI was the type of coping strategy. Confronting behavior was defined as either active confrontation of a height situation (“I actively expose myself to height situations”), or of planned or effective participation in height training (“Do you plan to participate in training classes to prevent vertigo caused by heights?”, “Did you participate in “training classes to prevent vertigo caused by heights”?”), or both. The questionnaire comprised questions on sociodemographic characteristics (age, sex, occupation, and education), subjective health status, and self-efficacy beliefs. Participants with vHI were asked to answer questions about symptoms, triggering situations, and coping strategies.

Self-efficacy was assessed with the German General Self-Efficacy scale (GSE) [30], [31], which measures general perceived self-efficacy. The GSE scale was developed on the basis of Bandura’s concepts. It has proven reliable and valid in various field studies and been translated into 28 languages [32].The scale includes ten items. A typical item is “Thanks to my resourcefulness, I can handle unforeseen situations.” Possible responses are not at all true (1), hardly true (2), moderately true (3), and exactly true (4), yielding a total score of 10 to 40. The mean total score of a norm sample of the German population was 29.4 [33].

General health was assessed by the general health question from the SF-12 questionnaire [34].

### Statistical analysis

Means were used for continuous variables and percentages for categorical variables. Explorative t tests and Chi-square tests were used for comparisons of individuals with and without vHI symptoms.

GSE is reported to show complex interactions with emotional, social, and cognitive functioning [35]. Aspects like depression, anxiety, and somatization may also be associated with the prevalence of vHI. In general, a covariate is considered a confounder if it is associated with, but not affected by, the exposure (in this study, GSE) and is a direct cause of the outcome (in this study, vHI). It is unproblematic to adjust for a confounder in multiple regression analysis. However, if covariates are intermediate factors or common effects of exposure and outcome [36], traditional adjustment by regression analysis will cause substantial bias, instead of reducing it. Recently, the use of directed acyclic graphs (DAG) was proposed in epidemiology in order to formalize causal association structures [37]. A DAG is a theoretical visualization of a whole causal network that links exposure and outcome. It consists of nodes representing variables (e.g., GSE, vHI, depression, sex, anxiety) and arrows representing causal associations between these variables. In a situation of complex interaction of variables, a DAG can be constructed based on previous knowledge, e.g. from literature or from experts. The graph can then be analyzed to determine the minimally sufficient adjustment set using predefined rules [38], [39]. A minimally sufficient adjustment set consists of the smallest number of variables needed to account for confounding. Adjustment of regression models for these sets allows us to estimate the association of exposure and outcome, here of GSE and vHI, in a less-biased way [40]. To ascertain whether the proposed association between GSE and vHI was affected by confounding, intermediate variables, or common effects, DAGs were used. The graph was based on characteristics with known association to GSE and vHI, specifically anxiety, depression, somatization, age, alcohol consumption and level of education. The resulting minimally sufficient adjustment set was then entered into a multiple logistic regression model to determine the effect of GSE and covariates on primary and secondary outcomes.

IBM SPSS Statistics Version 19 and SAS V9.3 (SAS Institute Inc., Cary, NC, USA) were used for statistical analyses. DAGitty [41] was used to construct and analyze DAGs.

## Results

Two of the initially contacted 1,166 individuals had died in the meantime. Twenty-five envelopes were returned unopened indicating that the addressee had relocated without leaving a forwarding address. Six hundred and sixteen (54%) individuals responded; of those, 607 (mean age 53.9, 50.3% were females) provided complete information on their vHI status (with vHI: mean age 53.9, 51.6% were females, without vHI: mean age 54.1, 47.5% were females).

Four hundred and seven participants reported life-time presence of vHI. Table 1 shows the characteristics of the participants, stratified by vHI status.

**Table 1:** Sociodemographic characteristics.

	N	total	vHI +	vHI -	p-value
**Age/years mean (SD)**	607	54.4 (13.3)	53.9 (13.3)	54.1 (15.9)	0.845
**Sex**	607				0.388
female		305 (50.2%)	210 (51.6%)	95 (47.5%)	
**education**	590				0.957
Grade school without vocational training		3 (0.5%)	2 (0.5%)	1 (0.5%)	
Grade school with vocational training		31 (5.3%)	22 (5.5%)	9 (4.7%)	
Secondary school		99 (16.8%)	64 (16.1%)	35 (18.2%)	
University entrance diploma, university		453 (76.7%)	307 (77.1%)	146 (76.0%)	
Still attending school		4 (0.7%)	3 (0.8%)	1 (0.5%)	
**Occupational situation**	607				0.086
employed		395 (65.1%)	274 (67.5%)	121 (60.2%)	
**general health^1^**	604				0.022
excellent		74 (12.3%)	49 (12.1%)	25 (12.5%)	
very good		301 (49.8%)	189 (46.8%)	112 (56.0%)	
good		201 (33.3%)	141 (34.9%)	60 (30.0%)	
fair		28 (4.6%)	25 (6.2%)	3 (1.5%)	
poor		0 (0%)	0 (0%)	0 (0%)	
**percieved self-efficacy mean (SD)**	605	31.8 (4.4)	31.4(4.3)	32.5 (4.3)	0.003

^1^ SF-12.

vHI+ =  participants with visual height intolerance.

vHI− =  participants without visual height intolerance.

Participants with vHI scored on average 31.4 points (SD 4.3) on the GSE scale, participants without vHI had an average score of 32.5 (SD 4.3). The difference was significant (df  = 603, t  = −2.95, p  = 0.003). Among individuals with vHI, 22.4% reported confronting behavior, i.e., actively confronting heights or intending to do height training. Confronters were significantly younger (df  = 201.15, t  = 4.07, p<.0001, 49.7 vs. 55.2 years), more likely to be female (df  = 1, chi^2^ = 8.3214, p  = 0.0039, 64.3% female), and on average scored higher on the GSE scale (df  = 402, t  = −2.79, p  = 0.0049, 32.5 vs. 31.1).

**Figure 1:**
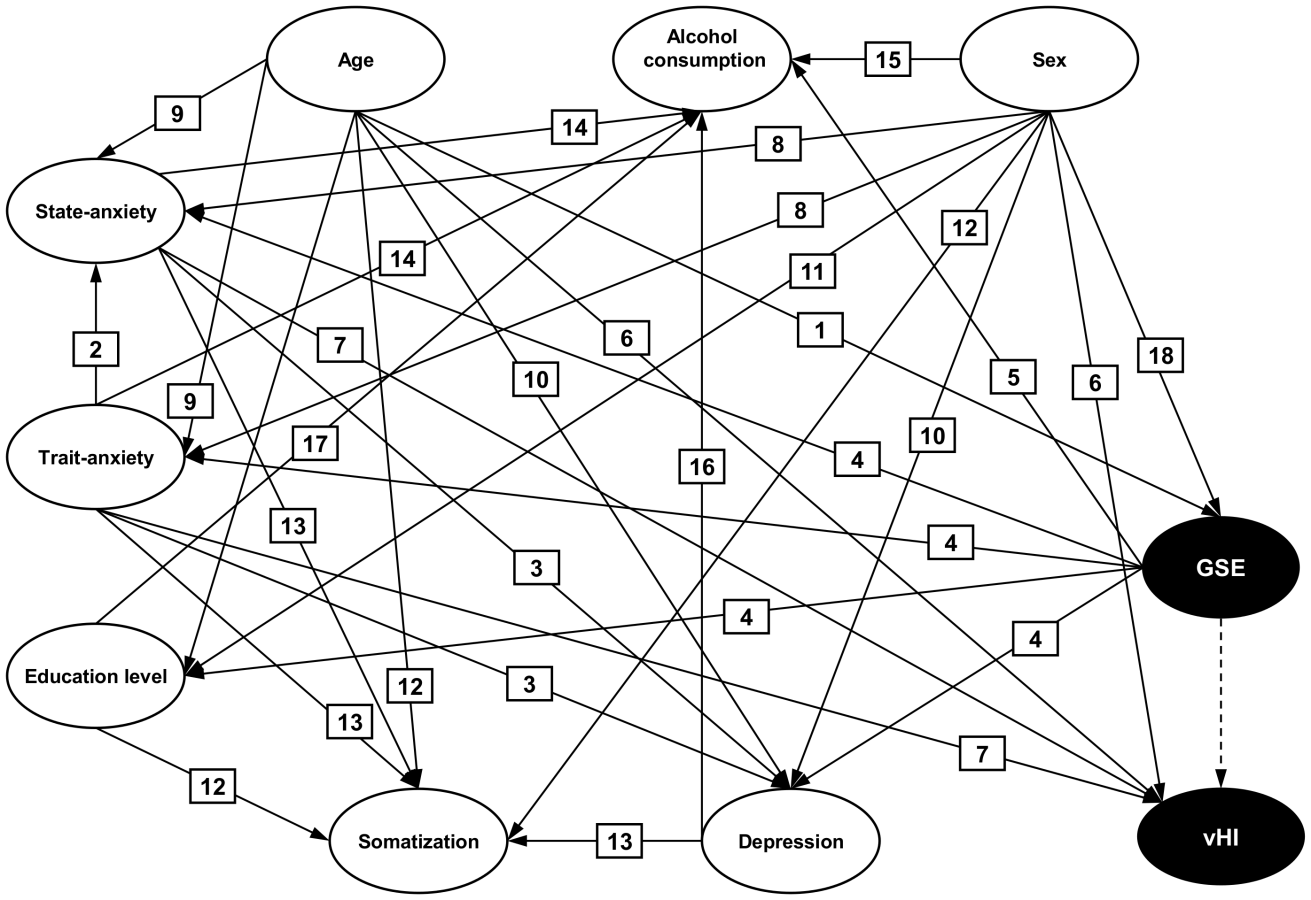
DAG derived from literature and expert knowledge - Nodes represent variables and arrows represent causal associations. Darkly colored nodes label exposure (general self-efficacy, GSE) and outcome (visual height intolerance, vHI). The dashed arrow indicates the postulated association between exposure and outcome. Numbers represent available sources of literature describing the associations. References for these associations are given in S1 Appendix.

Fig. 1 shows the DAG along with references describing the empirically confirmed association between two variables. The DAG algorithm identified a minimally sufficient adjustment set of GSE score and age. Sensitivity analyses with different DAG structures yielded similar sets. Entering the variables from the minimally sufficient adjustment set into logistic regression resulted in the significant association of GSE and vHI while adjusting for age (OR  = 0.95 for an increase of one point on the GSE scale, p  = 0.006, df  = 1). The effect of age was non-significant (OR  = 1.00, p  = 0.982, df  = 1). In the group of individuals with vHI, high GSE was an independent predictor for confronting behaviour (OR  = 0.92, p  = 0.003, df  = 1) while adjusting for age and sex. Also, younger persons were more likely to be confronters (OR  = 1.03 for an increase of one year of age, p  = 0.0016, df  = 1), as were women (OR  = 0.51, 0.0083, df  = 1).

## Discussion

The present study supports the theory that self-efficacy beliefs are associated with visual height intolerance (vHI). Persons with vHI were found to be less confident in their own competency to control challenging demands and threats. Also, those individuals who reported to actively confront their condition (confronters) had higher scores on the general self-efficacy scale than persons with vHI who avoided heights (avoiders).

These findings are in line with the literature. People with high self-efficacy were described as persevering to achieve their goals, using active rather than passive strategies for solving problems, and concentrating on opportunities rather than on obstacles [35]. Pain patients who are self-efficacious, i.e., believe that they will be able to cope, were found to be more active and less disabled by their pain [42]. The concept of general self-efficacy (GSE) examined in our study refers to perceived personal competence [32]. This competence may be specific to heights and to the correct interpretation of physical symptoms in the presence of heights [43].

The mean GSE score in our sample was found to be above the reported population norm values. This might be explained by characteristics like personality and physical fitness that are probably typical for members of a mountaineering association [44]. Thus it is not surprising that even the mean GSE of individuals with avoidant behavior in our study exceeded the population norm [33]. It should also be noted that the over-all difference in GSE score between persons with and without vHI was significant but not necessarily of a psychologically relevant magnitude. Therefore, benefit from addressing self-efficacy in training programs would likely be more apparent in individuals with low GSE. The difference between confronters and avoiders is specifically noteworthy because it confirms qualitative findings and points at potential for tailored therapy.

In previous representative studies vHI and acrophobia were significantly more prevalent in women than in men [2], [6]. Yet, the finding that women with vHI had a higher probability of confronting behavior was surprising. This is in line with our previous study where vHI was predictive of later panic attacks, agoraphobic fears and generalized anxiety only in men but not in women [6]. While the results of the present non-representative sample do not allow an explanation for this, the gender-specific role of self-efficacy in vHI merits further consideration.

In a situation of conflicting or strongly correlated concepts such as self-efficacy, depression, and anxiety, the results of the usual regression model can be misleading. Therefore, we used DAGs to disentangle those variables that might potentially bias the association of GSE and vHI. Although the development of a DAG can be time-consuming, it may be the only way to provide unbiased adjustment in some situations [45]. Structural equation modelling (SEM) would be an alternative to DAGs in an observational study with complex pathways. However, SEM works with strong distributional assumptions, while DAGs are nonparametric in nature and can therefore serve conceptual purposes rather than yield direct estimates of association [46]. A drawback of both DAG and SEM is that, while the presence of arrows is confirmed by evidence and plausibility, their absence is not directly acknowledged. Thus, potentially unmeasured confounding can bias the association. We addressed this challenge in our study by sensitivity analyses of different DAG representations.

The results of our study have implications that may be meaningful for the design of treatment and training programs. Our results suggest that vHI has several distinct constituents, some of which are somatic, and some cognitive. It has been postulated that acrophobia is the manifestation of visual field dependence and postural hypersensitivity [47]. Individuals with mainly somatic vHI may find it easy to confront their problem and to seek exposure therapy. The access to treatment of individuals with a predominance of cognitive components is less straightforward. For them, strategies might be warranted that increase general control and self-efficacy beliefs. This is in line with literature postulating that the effectiveness of exposure-based treatment may largely depend on cognitive factors [48]. The difference in self-efficacy between confronters and avoiders is a finding of our study that points at relevant strategies for therapy and training. Intervention studies will have to show if training to accommodate vHI is more effective if it not only dissuades clients of the dangerousness of the situation but also raises their feeling of self-efficacy [26].

The major limitation of this study is that our sampling procedure relied on an already established group of persons to increase response rates; all were members of the Alpine Association who had responded to a written questionnaire. Participants were likely to differ from a truly population-based sample. However, since we did not aim to give estimates for population-based prevalences but intended to examine contrasts, representativeness was not necessarily warranted. Due to the sampling strategy, participants were more homogeneous than a representative sample. Arguably this accounts for the low group difference in GSE. Lack of variance is thus bound to conceal associations and bias the result towards the null value. Therefore, any association shown in this study is likely to be more robust. Likewise, members of the Alpine Association might be more often exposed to situations that cause space and motion discomfort and be therefore more at risk to develop symptoms in presence of a unknown vestibular dysfunction [15], [49], [50]. The association of vHI and manifest vestibular dysfunction is still not completely understood and needs further research. Also, this study is a cross-sectional study without evidence on causality. Low self-efficacy may arise from the complex experience of lack of skill and of learning and be a consequence of a negatively experienced situation. One major drawback of our study is that we do not have information on the specific skill set of participants. Longitudinal and interventional studies are thus needed to clarify this association.

## Conclusions

Our study provides evidence for the association of perceived self-efficacy and visual height intolerance. We showed that low self-efficacy is predictive of not actively seeking help or confronting the height situation in persons with vHI. These findings may have consequences for strategies of alleviation and therapy of vHI.

## Supporting Information

S1 Appendix **References for the associations shown in** **Fig. 1** **.**
(DOCX)
