# Supplementary material for: Self-Efficacy Beliefs Are Associated with Visual Height Intolerance: A Cross-Sectional Survey
Source: PLoS One. 2014 Dec 30;9(12):e116220. doi: 10.1371/journal.pone.0116220 (PMC4280199; doi:10.1371/journal.pone.0116220)
Supplement: S1 Appendix — References for the associations shown in Fig. 1 . (DOCX) [file pone.0116220.s001.docx]

**Appendix 1.** References for the associations shown in figure 1

1. Artistico D, Cervone D, Pezzuti L (2003) Perceived self-efficacy and everyday problem solving among young and older adults. Psychology and Aging 18: 68-79
2. Bandura A (1994) Self-efficacy. In: S. Ramachaudran, editor. Encyclopedia of human behavior. New York: Academic Press. pp. 71-81
3. Schunk DH, Meece JL (2006) Self-efficacy development in adolescences. In: S. Pajares, T. Urdan, editors. Self-efficacy beliefs of adolescents*.* Greenwich: Information Publishing. pp. 71-96.
4. Spielberger CD (1972) Anxiety: Current trends in theory and research. New York: Academic Press.
5. Angst J (1996) Comorbidity of mood disorders: A longitudinal perspective study. Britisch Journal of Psychiatry 168: 31-37
6. Hautzinger M (1986) Differenzielle psychologische Theorien zu Angst und Depression. In: H. Helmchen, M. Linden, editors. Die Differenzierung von Angst und Depression. Heidelberg: Springer. pp. 123-134

Merikangas KS, Angst J, Eaton W, Canino G, Rubio-Stipec M, et al. (1996) Comorbidity and boundaries of affective disorders with anxiety disorders and substance misuse: Results of an International Task Force. British Journal of Psychiatry 168: 58-67.

Weissmann MM (1988) The epidemiology of anxiety disorders: Rates, Risks and familial patterns. J. psychiat. Res. 22: 99-114.

1. Bandura A (1993) Perceived Self-Efficacy in Cognitive Development and Functioning. Educational Psychologist 28: 117-148.
2. Oei TPS, Fergusson S, Lee NK (1998) The Differential Role of Alcohol Expectancies and Drinking Refusal Self-Efficacy in Problem and Nonproblem Drinkers. J. Stud. Alcohol 59: 704-711.
3. Huppert D, Grill E, Brandt T (2013) Down on heights? One in three has visual height intolerance. Journal of Neurology 260: 597–604.
4. Boffino CC, Cardoso de Sá CS, Gorenstein C, Brown RG, Basile LFH, et al. (2009) Fear of heights: cognitive performance and postural control. Eur Arch Psychiatry Clin Neurosci 259: 114-119.
5. Krasucki C, Howard R, Mann A (1998) The relationship between anxiety disorders and age. Int J Geriatr Psychiatry 13: 79-99.
6. Henderson AS, Jorm AF, Korten AE, Jacomb P, Christensen H, et al. (1998) Symptoms of depression and anxiety during adult life: evidence for a decline in prevalence with age. Psychological Medicine 28: 1321-1328.
7. Stordal E, Bjartveit Krüger M, Dahl NH, Krüger O, Mykletun A, et al. (2001) Depression in relation to age and gender in the general population: the Nord-Trøndelag Health Study (HUNT). Acta Psychiatr Scand 104: 210-6.
8. Alon S and Gelbgiser D (2011) The female advantage in college academic achievements and horizontal sex segregation. Social Science Research 40: 107–119.
9. Hiller W, Rief W, Brähler E (2006) Somatization in the population: from mild bodily misperceptions to disabling symptoms. Soc Psychiatry Psychiatr Epidemiol 41: 704-12.
10. Katon W, Sullivan M, Walker E (2001) Medical symptoms without identified pathology: relationship to psychiatric disorders, childhood and adult trauma, and personality traits. Ann Intern Med 134: 917-25.
11. Kushner MG, Sher KJ, Erickson DJ (1999) Prospective Analysis of the Relation Between DSM-III Anxiety Disorders and Alcohol Use Disorders. Am J Psychiatry 156: 723-732.
12. Nolen-Hoeksema S (2004) Gender differences in risk factors and consequences for alcohol use and problems. Gender Clin Psychol Rev 24: 981-1010.
13. Abraham HD, Fava M (1999) Order of onset of substance abuse and depression in a sample of depressed outpatients. Compr Psychiatry 40: 44-50.
14. Crum RM, Helzer JE, Anthony JC (1993) Level of education and alcohol abuse and dependence in adulthood: a further inquiry. Am J Public Health 83: 830-837.

Gilman SE, Breslau J, Conron KJ, Koenen KC, Subramanian SV, et al. (2008) Education and race-ethnicity differences in the lifetime risk of alcohol dependence. J Epidemiol Community Health 62: 224-230.

Malyutina S, Bobak M, Kurilovitch S, Nikitin Y, Marmot M (2004) Trends in alcohol intake by education and marital status in an urban population in russia between the mid 1980s and the mid 1990s. Alcohol and Alcoholism 39: 64-69.

1. Bussey K, Bandura A (1999) Social Cognitive Theory of Gender Development and Differentiation. Psychological Review 106: 676-713.
